# Comprehensive scoping review on adherence to 24-hour movement guidelines and socioeconomic indicators in children and adolescents

**DOI:** 10.1371/journal.pone.0321103

**Published:** 2025-04-17

**Authors:** Letícia Gonçalves, Suellem Zanlorenci, Melquesedek Ferreira Da Silva Almeida, Jérémy Vanhelst, Diego Augusto Santos Silva

**Affiliations:** 1 Department of Physical Education, Federal University of Santa Catarina-Sports Center, University Campus-Trindade-n. 476, Research Center in Kinanthropometry and Human Performance, Florianópolis, Santa Catarina, Brazil; 2 Université Sorbonne Paris Nord and Université Paris Cité, INSERM, INRAE, CNAM, Center of Research in Epidemiology and StatisticS (CRESS), Nutritional Epidemiology Research Team (EREN), Bobigny, France; University of Montenegro: Univerzitet Crne Gore, MONTENEGRO

## Abstract

**Background:**

Adherence to the 24-hour movement behavior guidelines has been used to identify potential impacts on health indicators in children and adolescents. However, information on the association between socioeconomic indicators and adherence to the guidelines remains unclear.

**Objective:**

The scoping review aims to identify and synthesize scientific evidence on the associations between socioeconomic indicators and adherence to the 24-hour movement behavior guidelines in children and adolescents.

**Methods:**

A systematic search was conducted in PubMed, Web of Science, Scopus, SPORTDiscus, SciELO, CINAHL, and EMBASE. Studies were selected if they included a population of children and adolescents aged 5–17 years and addressed the relationship between adherence to the 24-hour movement behavior guidelines and socioeconomic indicators.

**Results:**

From 1,871 articles identified, 10 studies with data from 562,505 children and adolescents across 10 countries were included. Self-reported questionnaires were the most common measurement method for variables related to the 24-hour movement behaviors (n=6). The Canadian 24-Hour Movement Guidelines were the most frequently used reference for classifying target behaviors (n=4). Socioeconomic indicators at the individual and/or family level were used in most investigations, specifically parental education (n=7) and household income (n=6). Most findings were inconclusive regarding the relationship between adherence to the 24-hour movement behavior guidelines and socioeconomic indicators.

**Conclusions:**

Studies on this interrelation have been limited, with inconclusive results regarding associations between socioeconomic indicators and adherence to the 24-hour movement behavior guidelines in children and adolescents. Further research is needed to better understand these relationships.

## Introduction

Twenty-four-hour movement behaviors are defined as the integration of variables that encompass sleep, sedentary behavior, and physical activity, distributed across the entire day [1,2]. Investigating these behaviors—specifically high levels of physical activity, low levels of sedentary behavior, and optimal sleep duration – has been widely used to identify and understand their potential effects on positive changes in health indicators among children and adolescents. These health indicators include reductions in reduced adiposity [3,4], improved bone, skeletal, and cardiometabolic health, as well as social and emotional factors [4], and a lower risk of mortality [3].

According to the Canadian 24-Hour Movement Guidelines [1,2], children and adolescents aged 5–17 years should engage in at least 60 minutes of moderate to vigorous physical activity daily, including a variety of aerobic activities. Additionally, muscle- and bone-strengthening activities should be incorporated at least three days per week. The guidelines also recommend several hours per day of light physical activities, both structured and unstructured [1,2]. For sedentary behavior, a limit of no more than 2 hours per day of recreational screen time is advised, along with minimizing prolonged sitting periods [1,2]. Furthermore, uninterrupted sleep is recommended: 9–11 hours per night for children aged 5–13 years, and 8–10 hours per night for adolescents aged 14–17 years, with consistent bedtimes and wake-up times [1,2].

Although the contribution of 24-hour movement behaviors to overall health is well established [5,6], a review across 23 countries estimated that 19.21% of children and adolescents aged 3–18 did not meet the recommendations between 2016 and 2021 [7]. Likewise, the joint association of these behaviors with socioeconomic indicators has been minimally explored [7]. Evidence shows that children and adolescents with low socioeconomic status are generally less physically active than their higher socioeconomic status counterparts [8,9]. Additionally, an increasing body of research indicates that low socioeconomic status is associated with poorer perceived sleep quality, shorter sleep duration, less consistent sleep patterns throughout the week, and greater daytime sleepiness [10,11]. In high-income countries, socioeconomic status has been inversely associated with sedentary behavior, whereas in low- and middle-income countries, socioeconomic status shows a positive association with sedentary behavior [12].

Several previously published literature reviews have examined the relationship between socioeconomic indicators and various health outcomes in children and adolescents [8,13–16]. In summary, most of these reviews have shown that the better the investigated socioeconomic indicators, the better the health outcomes [8,13–16]. Regarding the association between socioeconomic indicators and the set of 24-hour movement behaviors, no single direction appears to prevail. This is likely because most studies have focused on only one movement behavior (physical activity, or sedentary behavior/screen time, or sleep) [8,13–16], which limits understanding, as results may differ when considering the combined and simultaneous association of all three movement behaviors.

Existing review on this topic is available [7] but have primarily focused on a limited range of socioeconomic variables, especially contextual ones (i.e., Human Development Index and geographic location of countries/regions). Additionally, the influence or heterogeneity of results based on individual-level socioeconomic indicators (e.g., family income, family economic status, parental education level, and housing) and contextual indicators (e.g., region of residence, area of residence, socioeconomic deprivation, etc.) has not been adequately explored in the assessment of their association with 24-hour movement behaviors across studies. Identifying and synthesizing evidence regarding the association between this set of behaviors and socioeconomic indicators could help establish priorities, guide public health policies, and develop strategies that promote adherence to the 24-hour movement behavior guidelines. Furthermore, given the complexity and interdependence of these behaviors [2], investigating these interrelationships could identify subgroups more disadvantaged in terms of adherence, thus facilitating the creation of more effective and targeted interventions.

Therefore, the objective of this scoping review is to identify and synthesize scientific evidence on the associations between socioeconomic indicators and adherence to the 24-hour movement behavior guidelines in children and adolescents aged 5–17. The hypothesis of this study is that socioeconomic indicators significantly influence adherence to the 24-hour movement behavior guidelines in children and adolescents, with better socioeconomic conditions being associated with greater adherence to the guidelines.

## Materials and methods

### Protocol and checklist

The present scoping review was conducted following the PRISMA-ScR checklist [17] and the Joanna Briggs Institute Reviewers’ guidelines [18]. The final protocol for this scoping review was previously registered on the Open Science Framework (OSF) platform (https://osf.io/bhx4s, accessed on July 18, 2024). This review followed six steps recommended by JBI: 1) identification of the research question; 2) screening of evidence related to the topic; 3) selection of evidence; 4) analysis of information; 5) grouping, synthesis, and presentation of information/data. The sixth step, considered optional, will not be utilized (i.e., assessment of the quality of the risk of bias).

### Review questions

The formulation of the research question was guided by the “Population”, “Concept”, and “Context” elements – (PCC) [18]. Accordingly, the following definitions were established for this review: P – children and adolescents aged 5–17 years, as the 24-hour movement behavior guidelines target this age group [2]; C – evidence related to adherence to the 24-hour movement behavior guidelines, which encompass a combination of physical activity, sedentary behavior, and sleep; C – evidence related to individual-level socioeconomic indicators (e.g., family income, family economic status, parental education level, housing, etc.) and contextual indicators (e.g., region of residence, area of residence, socioeconomic deprivation, etc.). Based on these elements, the following research question was defined: *What is the scientific evidence on the associations between socioeconomic indicators and adherence to the 24-hour movement behavior guidelines in children and adolescents aged 5–17 years?*

### Inclusion and exclusion criteria

Eligible studies for this review included: original research published in scientific journals, whether quantitative or qualitative; studies that considered information on adherence to the 24-hour movement behavior guidelines and be conducted with children and adolescents aged 5–17 years, without any diagnosed diseases or special clinical conditions; and publications in any language. The primary outcomes of interest were studies that examined the association between individual-level and contextual socioeconomic indicators and adherence to the 24-hour movement behavior guidelines.

The exclusion criteria for this review were as follows: a) studies published prior to 2015, as original articles analyzing 24-hour movement behaviors emerged in 2015, and the first 24-hour movement behavior guidelines were launched in 2016 [2]; b) articles with objectives unrelated to the present review (not addressing evidence related to the PCC elements); c) dissertations, theses, book chapters, conference abstracts and presentations, opinion articles, methodological articles, and reviews; d) studies not available in full in the investigated data sources, even after contacting the authors via email.

### Databases and search strategy

Evidence searches were conducted in July 2024 and adapted for application across all databases based on the method developed for PubMed, using a combination of terms for 24-hour movement behavior, socioeconomic indicators, and children and adolescents. The descriptors for each of these terms were identified from studies referenced in the literature [4,7,19], as well as consultations with the Medical Subject Headings (MeSH) platform and Health Sciences Descriptors (DECS). Descriptors in Portuguese, English, and Spanish were included in seven electronic databases (PubMed, Web of Science, Scopus, SPORTDiscus, SciELO, CINAHL, and EMBASE), tailored to each platform’s search requirements. Additional details regarding the search strategy are available in S1 File. Manual searches were also conducted in the reference lists of included studies and in review articles that analyzed topics similar to those of this review. Additionally, experts in the field were contacted to identify any further relevant studies.

### Selection and evaluation of studies

Two independent researchers performed the study screening in each database, beginning with title and abstract review, followed by full-text examination of the studies selected based on inclusion/exclusion criteria. During data extraction, any discrepancies between the two researchers regarding study inclusion or exclusion were resolved by a third researcher. The search results were exported to the Rayyan software (Intelligent Systematic Review), a tool for screening, duplicate identification, and data extraction in a blinded system. In addition, the reference lists of the selected studies were examined to identify any additional studies relevant for potential inclusion in this review.

### Extraction and synthesis of information

Data from each study were extracted and organized using Microsoft^®^ Excel (Microsoft 2010, Version 14.7.7). First, descriptive information was gathered about each selected study, including study location, data collection year, study design, sample characteristics (sample size, sex, and age range), study objectives, author, and publication year. Additionally, specific characteristics of the selected studies were also recorded, such as tests/instruments used, classification applied for 24-hour movement behaviors, socioeconomic indicators, statistical analysis, and main findings.

Subsequently, methods for measuring 24-hour movement behaviors were identified, including device-based measurements, questionnaires, or a combination of both. Information on the socioeconomic indicators used in relation to 24-hour movement behaviors was also collected. Finally, the relationship between adherence to 24-hour movement behaviors and socioeconomic indicators was extracted, including information on the measurement methods used for target behaviors (device-based measurements, questionnaire-based measures, or both), type of socioeconomic indicator (individual or contextual), and the observed relationship outcome (positive, negative, or null association).

### Analysis of evidence and presentation of results

The review results were presented in a flowchart format, following JBI and PRISMA-ScR recommendations [17,18] aligning with the review’s objective. Additionally, tables were employed to illustrate the results, providing an evidence-based description of whether associations between 24-hour movement behaviors and socioeconomic indicators were identified, as well as detailing the methods used in each study.

## Results

### Search

The initial search across the seven databases yielded 1,871 articles. After removing duplicates (n=724), the titles and abstracts of 1,147 articles were screened. Of these, 1,089 were excluded for not meeting the inclusion criteria. Consequently, 58 articles were considered eligible for full-text review. Among these, 49 were excluded for failing to meet eligibility criteria, resulting in nine articles for this review. Following an additional review of the reference lists of the included articles, one more article was selected. As a result, a total of 10 studies were included in the scoping review ().

**Fig 1:**
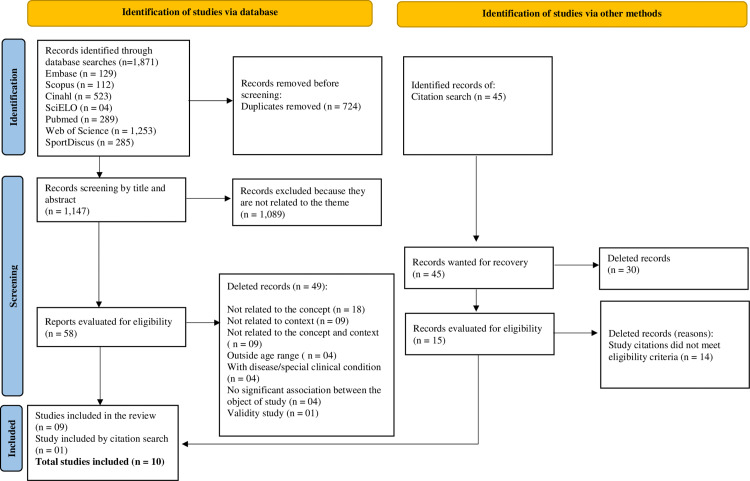
Search databases. Flowchart of searches for documents in this scoping review.

### Characteristics of the studies

These studies provided results from 562,505 participants across 10 countries, including Saudi Arabia [20], China [21,22], Mozambique [23], New Zealand [5], South Korea [24], the United Kingdom [25], the United States [26], Brazil [27], and Germany [28]. The data collection period across studies ranged from 2009 [5] to 2022 [28]. Of the included studies, nine were cross-sectional, and one was longitudinal [5], including children with an average age ranging from 6.5 ± 1.1 years [26] to 14.8 ±1.6 years [24]. Regarding sample size, the smallest study included 623 participants [5], while the largest included 114,072 participants [21]. All studies investigated both male and female children and adolescents (n=10).

Regarding the objectives of the studies, it was observed that eight studies (n=8) investigated the prevalence of adherence to the 24-hour movement guidelines, key correlates, as well as group differences [5,21–23,25–28] which included measures of body adiposity [21,25,26], residence location [23], sociodemographic correlates [5,22,26] and regional socioeconomic deprivation [28]. Two of the studies included in this review collected data during the COVID-19 public health emergency [20,28]; and one study analyzed a six-year trend and the intersectional correlates of adherence to the 24-hour movement guidelines [24] (S1 Table).

### Specific characteristics of the selected studies

#### Measurement methods for 24-hour movement behaviors.

Self-reported questionnaires were the most commonly used method for measuring variables related to 24-hour movement behaviors (physical activity, sedentary behavior/screen time, and sleep), appearing in six studies (n=6) [20–22,24,27,28]. Among these, five studies collected self-reported data directly from children or adolescents [21,22,24,27,28], while in one study, parents or guardians provided responses [20] (Table 1 and S2 Table).

Additionally, four studies were identified that combined two measurement methods (accelerometer-based motion devices and self-reported questionnaires) for assessing 24-hour movement behaviors, using either the accelerometer or self-reports for at least one target behavior [5,23,25,26]. Of these, three studies used accelerometers to measure two variables, including physical activity and sleep [5,23,26], while one study used accelerometers exclusively for physical activity [25]. These studies also varied in the model and placement of the device, with two studies using wrist-worn accelerometers [25,26], one using a hip-worn accelerometer [23], and one study utilizing accelerometers placed on the lower back and thigh [5]. Regarding the self-report method, two studies collected responses directly from children or adolescents [23,25], while in two studies, responses were provided by parents or guardians [5,26] (Table 1 and S2 Table).

**Table 1:** Number of studies that measured 24-hour movement behaviors using a questionnaire and an accelerometer.

	Questionnaire		Accelerometer		
	Children/Adolescents	Parents/caregivers	Hip	Wrist	Thigh and lower back
Physical Activity			1 [23]	2 [25,26]	1 [5]
Physical Activity Sedentary Behavior or Screen Time	2 [23,25]	2 [5,26]			
Sleep	1 [25]		1 [23]	1 [26]	1 [5]
24h MB	5 [21,22,24,27,28]	1 [20]			

24h MB: 24-hour Movement Behaviors.

#### Classification used to assess 24-hour movement behaviors.

Regarding the classification used to assess 24-hour movement behaviors, four studies were identified that reported using the Canadian 24-Hour Movement Guidelines (n=4) [21,22,24,28]. One study reported using two sets of guidelines, including the Canadian 24-Hour Movement Guidelines and the World Health Organization’s 2020 recommendations on physical activity and sedentary behavior (n=1) [20]. Another study reported using New Zealand’s 24-Hour Movement Guidelines (n=1) [5], while one study applied the Asia-Pacific 24-Hour Movement Guidelines (n=1) [26]. Two studies did not specify the recommendations used (n=2) [23,25] and one study applied specific recommendations for each variable investigated [27], including physical activity [29], screen time [30], and sleep [31] (S2 Table).

### Socioeconomic indicators

#### Measured at the individual and/or family level.

Regarding the socioeconomic indicators measured at the individual and/or family level in the included studies (i.e., assessing the social and economic situation of a person, considering individual aspects), parental education was the most commonly investigated indicator, appearing in seven studies [5,20–24,26]. This was followed by sixstudies examining annual income and/or income tertile [5,21,22,25,26]. Two studies explored parental employment status and/or weekly working hours [5,23], family composition [5,21], socioeconomic status [24,27], and social class [24]. One study investigated the number of televisions in the household [23], and another study examined the number of functional cars in the home [23]. Additional details on these indicators are available in Table 2.

**Table 2:** Number of studies by socioeconomic indicator analyzed in relation to 24-hour movement behaviors in children and adolescents.

Socioeconomic indicators	Number of studies^*^	Authors
**Measured at the individual and/or family level**		
Parents’ education	7	[5,20–24,26]
Income	6	[5,20–22,25,26]
Parents’ employment	2	[5,23]
Socioeconomic status	3	[5,24,27]
Social class	1	[24]
Family composition	3	[5,21,23]
Number of TVs in the home	1	[23]
Number of cars in the home	1	[23]
**Measured at the context level**		
Place of residence (urban/rural)	3	[5,21,22]
Region of residence	2	[20,27]
Location of school	1	[23]
Neighborhood crime rate	1	[23]
Regional deprivation index	1	[5]
Income of jurisdiction	1	[26]
Regional socioeconomic deprivation	1	[28]

*The same study may have measured more than one socioeconomic indicator.

#### Measured at the contextual level.

Considering socioeconomic indicators measured at the contextual level in the included studies (i.e., assessing social and economic aspects of a population or environment based on the specific context being analyzed), three studies included residence location (urban/rural) [5,21,22]. Two studies examined the geographical region of residence: one study conducted in Brazil stratified data across five geographic regions (North, Northeast, Southeast, South, and Central-West) [27], while another study in Saudi Arabia used stratification across 13 province [20]. Additionally, one study included school location [23], neighborhood crime rate [23], regional deprivation index [5], jurisdictional income level [26], and regional socioeconomic deprivation [28]. Further information on these indicators is provided in Table 2.

### Statistical analysis used in the simultaneous combination of variables

Of the studies analyzed, eight utilized multivariable models [21–28] and two used bivariate models in their statistical analyses [5,20] to examine the association between adherence to 24-hour movement behaviors and socioeconomic indicators (S2 Table).

### Results of selected studies

Based on the evidence summarized in this review and presented in Tables 3 and 4, 24-hour movement behaviors were primarily measured using two types of instruments: accelerometer-based motion devices and self-reported questionnaires. The socioeconomic indicators in the reviewed studies were measured at either the individual and/or family level or at the contextual level.

**Table 3:** Association between 24-hour movement behaviors and socioeconomic indicators of the studies analyzed.

Socioeconomic indicators	All 24h MB measured by questionnaire	Two 24h MB measured by questionnaire and one 24h MB measured by accelerometer	One 24h MB measured by questionnaire and two 24h MB measured by accelerometer
**Measured at the individual and/or family level**			
Parents’ education	– + 0 + [20–22,24]		+ – – [5,23,26]
Income	– + 0 [20–22]	+ [25]	0 – [5,26]
Parents’ employment			0 0 [5,23]
Socioeconomic status	+ 0 [24,27]		0 [5]
Social class	+ [24]		
Family composition	0 [21]		0 0 [5,23]
Number of TVs in the home			0 [23]
Number of cars in the home			0 [23]
**Measured at the context level**			
Place of residence (urban/rural)	+ – [21,22]		0 [5]
Region of residence	0 – [20,27]		
Location of school			– [23]
Neighborhood crime rate			0 [23]
Regional deprivation index			0 [5]
Income of jurisdiction			– [26]
Regional socioeconomic deprivation	– [28]		

+: directly proportional association; –: inversely proportional association; 0: null association; 24h MB: 24-hour Movement Behaviors

**Table 4:** Number of studies reporting positive, negative or null association between socioeconomic indicators and 24-hour movement behaviors.

Socioeconomic indicators	Association with 24-hour movement behaviors			Total studies
	Positive	Negative	Null	
**Measured at the individual and/or family level**				
Parents’ education	3	3	1	7
Income	2	2	2	6
Parents’ employment	0	0	2	2
Socioeconomic status	1	0	2	3
Social class	1	0	0	1
Family composition	0	0	3	3
Number of TVs in the home	0	0	1	1
Number of cars in the home	0	0	1	1
**Measured at the context level**				
Place of residence (urban/rural)	1	1	1	3
Region of residence	0	1	1	2
Location of school	0	1	0	1
Neighborhood crime rate	0	0	1	1
Regional deprivation index	0	0	1	1
Income of jurisdiction	0	1	0	1
Regional socioeconomic deprivation	0	1	0	1

Positive: higher chances; Negative: lower chances; Null: no association

Of the seven studies that investigated parental education as a socioeconomic indicator, three reported a positive association with 24-hour movement behaviors (i.e., as parental education increased, the likelihood or prevalence of adherence to the 24-hour movement behavior guidelines also increased, and vice versa) [5,21,24], while three studies reported a negative association with adherence to the guidelines (i.e., as parental education increased, the likelihood or prevalence of adherence to the 24-hour movement behavior guidelines decreased, and vice versa) [20,23,26]. Among the studies showing a positive association, two measured all movement behaviors using self-reported questionnaires [21,24], and one study used a questionnaire to measure one of the movement behaviors and an accelerometer to measure the other two target behaviors [5] (Tables 3 and 4).

Of the six studies that investigated income as a socioeconomic indicator, two reported a positive association with 24-hour movement behaviors (i.e., as income increased, the likelihood or prevalence of adherence to the 24-hour movement behavior guidelines also increased, and vice versa) [21,25]. Conversely, two studies reported a negative association with adherence to the 24-hour movement behavior guidelines (i.e., as income increased, the likelihood or prevalence of adherence to the 24-hour movement behavior guidelines decreased, and vice versa) [20,26]. Among the studies showing a positive association, one measured all movement behaviors using a self-reported questionnaire [21], while the other used a questionnaire to measure two of the movement behaviors and an accelerometer device to measure the remaining behavior [25] (Tables 3 and 4).

Of the three studies that investigated the socioeconomic status indicator, only one found a positive association with 24-hour movement behaviors (i.e., participants with higher socioeconomic status had greater chances of meeting the 24-hour movement behavior guidelines compared to those with lower socioeconomic status). This study assessed all 24-hour movement behaviors using a self-reported questionnaire [24] (Tables 3 and 4).

Regarding socioeconomic indicators measured at the contextual level, among the three studies that investigated residence location (urban/rural), one study found a positive association with 24-hour movement behaviors (i.e., those residing in urban areas had higher chances of meeting the 24-hour movement behavior guidelines compared to those in rural areas, with the authors’ reference category being urban regions) [21], while another study presented a negative association with adherence to the 24-hour movement behavior guidelines [22]. The study that showed a positive association between the variables measured all movement behaviors using a self-reported questionnaire (Tables 3 and 4) [21].

Additionally, one study that investigated the socioeconomic indicator of residential regions reported a negative association with 24-hour movement behaviors measured by a self-reported questionnaire (i.e., those living in more developed regions had lower chances of meeting the 24-hour movement behavior guidelines, with the authors’ reference category being developed regions) [27]. Another study examined regional socioeconomic deprivation [28] and also reported a negative association with 24-hour movement behaviors measured by self-reported questionnaire (i.e., individuals residing in more deprived areas had lower chances of meeting the 24-hour movement behavior guidelines compared to those from wealthier regions).

One study that investigated school location as a socioeconomic indicator reported a negative association with adherence to 24-hour movement behaviors (i.e., participants from rural schools were more likely to meet the 24-hour movement behavior guidelines compared to those from urban schools, with the reference category defined by the authors as urban regions) [23]. In this study, two of the targeted 24-hour movement behaviors were measured by accelerometer, and the third behavior was assessed by questionnaire [23]. Additionally, the study that investigated jurisdictional income [26] reported a negative association with 24-hour movement behaviors (i.e., the higher the jurisdictional income, the lower the likelihood of meeting the 24-hour movement behavior guidelines), with two of the target behaviors measured by accelerometer and the third by questionnaire (Tables 3 and 4).

All studies that investigated the socioeconomic indicators of parental employment, family composition, number of TVs at home, number of cars at home, neighborhood crime rate, and regional deprivation index found no association between these indicators and 24-hour movement behaviors (Tables 3 and 4).

## Discussion

This scoping review synthesized evidence from 10 studies that aimed to examine the association between socioeconomic indicators and adherence to 24-hour movement behavior guidelines in children and adolescents aged 5–17 years. The main findings of this review were as follows: (a) most studies employed self-reported questionnaires to assess all 24-hour movement behaviors, with responses provided directly by children or adolescents; (b) the majority of studies used the Canadian 24-Hour Movement Guidelines for classifying 24-hour movement behaviors; (c) to examine associations with 24-hour movement behaviors, most studies included socioeconomic indicators measured at the individual and/or family level, specifically parental education and income; (d) although few studies investigated additional socioeconomic indicators, the family composition was a variable for which all studies reported no association with 24-hour movement behaviors; (e) the studies included in this review found no association between the main socioeconomic indicators analyzed at the individual/family level, such as income and parental education, and 24-hour movement behaviors; (f) few studies included in this review collected data during the COVID-19 public health emergency.

Regarding the evaluation of tests/instruments for 24-hour movement behaviors, most studies employed self-reported questionnaires, a method known for its validity, reliability, low cost, and ability to capture contextual information (i.e., individuals report where, with whom, etc.) as well as retrospective data, thus allowing for the analysis of these behaviors over extended periods in large-scale studies [32]. However, this tool, like others used in these studies, has certain limitations, particularly its susceptibility to memory biases (i.e., children may have difficulty recalling durations and might overestimate or underestimate certain behaviors) [33,34]. Additionally, it is important to note that relevant factors, such as age differences (younger individuals may still be developing their concept of time, which can affect the validity of questionnaires) [32], sex, and parental education level [34], were not adequately reported in the methods of most studies. This may have contributed to the underestimation or overestimation of results. Therefore, adopting additional strategies to analyze 24-hour movement behaviors and socioeconomic indicators measured via questionnaires should be considered, as this could enhance the generalizability of findings across different cultural, ethnic, and economic contexts.

The studies included in this scoping review indicated that the Canadian 24-Hour Movement Guidelines were used as the reference for classifying the movement behavior variables analyzed. This finding may be explained by the fact that these guidelines were the first to be developed with a 24-hour movement approach, are widely recognized, and offer a holistic view of behaviors compared to various separate guidelines [35]. This approach facilitates appropriate comparisons and analyses, as these variables are interdependent (i.e., a change in one behavior often occurs at the expense of others) [35]. However, while the guidelines include evidence based on diverse populations and the interrelationship with health indicators [35], they also present limitations, as they can be influenced by various socioeconomic, cultural, and other correlates (e.g., age, sex, race, etc.) [36] which may pose barriers to adherence and are sometimes generalized and addressed in a decontextualized manner. Therefore, future studies are encouraged to consider evidence in a complex, individualized, and integrated way [36], with adaptations based on different contexts (specifically when investigating socioeconomic indicators and their relationship with 24-hour movement behaviors) [36], as it is likely that the associations identified may vary depending on how these parameters are approached.

This review found that parental education was the most frequently investigated socioeconomic indicator in relation to 24-hour movement behaviors. It is suggested that this parental education may directly impact the adoption of these movement behaviors, as parents with higher education levels tend to have greater knowledge, which can positively influence [37,38] the adoption of preventive health behaviors in children and adolescents, such as regular physical activity, adequate sleep, and reduced sedentary time [38,39]. Conversely, parents with lower education levels may face barriers related to a lack of knowledge about the promotion of these healthy behaviors and may be less likely to influence their children to adopt them [40]. Parental education level has been widely used among studies and shown to be a valuable indicator of the influence of socioeconomic disparities on promoting healthy behaviors [38] . However, the adoption of healthy behaviors is complex, and more studies utilizing other socioeconomic indicators are needed, as the magnitude of associations may vary depending on the socioeconomic indicator and the context investigated [41].

Another socioeconomic indicator identified in this review in association with 24-hour movement behaviors in children and adolescents was family income. The relationship between these variables appears to be significant, as family income is an independent predictor of variables related to 24-hour movement behaviors or overall health [38]. That is, as income increases, individuals are likely to experience less exposure to negative psychosocial factors (e.g., stress, insecurity, future concerns) and have greater access to financial resources that create a more supportive environment (e.g., housing, healthcare) [38] for adopting these healthy behaviors [38,42]. In this context, although the studies included in this review did not identify a consensus regarding the relationship between family income and adherence to 24-hour movement behavior guidelines, this may be due to the varying methods of income measurement across the studies. These differences include varied data analyses (multivariable approaches [21,22,25,26] versus bivariate approaches [5] and different categorizations of income, despite all studies including questions to capture this variable. These methodological differences in income treatment could contribute to the lack of consistency in the findings.

The results described in the literature and included in this review did not report an association between adherence to 24-hour movement behavior guidelines and family composition across all studies analyzed. The plausibility for this lack of association may be related to the fact that family composition alone may not be a sole determinant in the adoption of healthy behaviors; rather, it is the modulating role of the family (i.e., regardless of family structure) that influences the adoption of 24-hour movement behaviors. This includes family social support, which contributes to the adoption of these behaviors (e.g., encouragement, engagement, logistical support, and parenting style) [43–46]. Therefore, these results should be interpreted with caution, as the significant effects of caregiver influence were not considered in the analyzed studies.

Although most studies described in the literature and reported in this review did not reach a consensus on the association between different socioeconomic indicators and 24-hour movement behaviors, certain caveats should be considered, as studies showed divergent results for the same indicators. These include: (a) methodological differences between studies investigating 24-hour movement behaviors, with some using device-derived methods [5,23,25,26] and others using questionnaires [21,22,24,27,28] for the same variables; (b) a lack of standardization in measurement instruments and an absence of references for classifying socioeconomic variables, which reflects heterogeneous inclusion of these aspects in the context of a literature review and may have contributed to the inconsistency in results; (c) another factor that could explain the inconsistent results is the lack of strategies to moderate or mediate the analyzed relationship, which would allow for a better interpretation of the obtained results. A larger body of evidence is suggested to confirm the direction of these associations, considering that, beyond directly impacting adherence to 24-hour movement behavior guidelines [5,21–28], socioeconomic indicators have been directly linked to general living and health conditions [47,48].

Two of the studies included in this review collected data during the COVID-19 public health emergency [20,28]. The study by Alanazi et al. (2022) aimed to assess the impact of pandemic restrictions on 24-hour movement behaviors, and according to the authors’ findings, adherence to 24-hour movement behavior decreased as parental education and income levels increased. The study by Suchert et al. (2023) aimed to examine the relationship between regional socioeconomic deprivation and adherence to the 24-hour movement guidelines among children and adolescents. The authors found adolescents witch in highest socioeconomic deprivation were less likely to meet the 24-hour movement guidelines when compared to adolescents from richer regions. Although the findings from pre-pandemic studies indicated either similar or divergent patterns compared to those conducted during the pandemic, this review highlights that the COVID-19 pandemic led to changes both in socioeconomic indicators for many families worldwide, who faced job losses and salary cuts [49], as well as in the daily routines of children and adolescents, potentially affecting their physical activity, screen time/sedentary behavior, and sleep habits [20].Therefore, this review is comprehensive enough to include information from the entire body of literature, even those studies conducted during a critical period for humanity, such as the COVID-19 pandemic.

The strengths of this review include the extensive number of databases and amount of information analyzed, as well as the structured presentation of results according to tests and instruments, classifications, various socioeconomic indicators, and statistical analyses used in the reviewed studies. However, certain limitations should be noted, such as the limited number of studies with the primary objective of investigating the association between 24-hour movement behaviors and socioeconomic indicators, which reduces the ability to establish conclusive associations. Although a rigorous methodological approach was applied in the information search, gray literature was not considered, as it may present methodological weaknesses in study design and reliability of findings [50]. Furthermore, although the evidence reported by the studies did not indicate a clear direction, there is limited information available in the literature regarding the interrelationship of 24-hour movement behaviors and socioeconomic indicators, contributing to the inconclusive nature of these associations. Additionally, most of the evidence is derived from cross-sectional studies, which prevents inferences about causality and directionality. Longitudinal studies are needed, as they could offer insights into the role of 24-hour movement behaviors in children and adolescents and potential variations based on socioeconomic indicators over time. Additionally, comparing studies that used different measurement instruments for socioeconomic indicators, whether at the individual or contextual level, posed a challenge for this review. Depending on the instrument employed, the aspects considered may vary, which can influence the association with 24-hour movement behaviors. Such heterogeneity can be viewed as a limitation of the present review. Lastly, it is important to note that most methods and instruments used for assessing 24-hour movement behaviors in children present limitations. In particular, both accelerometers and questionnaires, widely used for this purpose, face methodological challenges such as variability in calibration, the need for standardized usage protocols, and potential inaccuracies in subjective reporting. These limitations may undermine the precision and comparability of results, posing an obstacle to more robust and reliable assessments of these behaviors.

## Conclusion

Based on the findings of this scoping review, it can be concluded that the results of the included studies were inconclusive regarding the relationship between socioeconomic indicators at the individual/family level and/or the contextual level and adherence to 24-hour movement behaviors, primarily because these aspects have been underreported in the literature. In terms of methods for assessing 24-hour movement behaviors, self-reporting—directly completed by children or adolescents—was commonly used. To classify 24-hour movement behaviors, most studies applied the Canadian 24-Hour Movement Guidelines. Furthermore, the majority of studies in this review included socioeconomic indicators measured at the individual and/or family level, specifically parental education and income, in examining associations with 24-hour movement behaviors. Additional findings from this review indicated that, although few studies investigated family composition as a socioeconomic indicator, all reported a null association with 24-hour movement behaviors.

Future research in this area should delve deeper into socioeconomic indicators at the contextual level, such as neighborhood characteristics and access to community resources, which remain underexplored in the literature. Expanding the scope of socioeconomic indicators beyond parental education and income is also crucial to capture a broader understanding of social influences on adherence to these behaviors. Research in culturally and economically diverse contexts is another priority to better understand how socioeconomic conditions influence movement behaviors across populations. Longitudinal studies are also recommended to provide insights into how movement behaviors change over time in response to shifts in socioeconomic indicators at both individual and contextual levels. Lastly, it is important to explore how 24-hour movement guidelines, such as the Canadian guidelines, can be adapted to different socioeconomic and cultural contexts to enhance adherence among vulnerable populations.

## Supporting information

S1 Table Description of the characteristics of the selected studies. (PDF)

S2 Table Specific characteristics of selected studies. (PDF)

S1 File Search strategy databases. (PDF)
