# Supplementary material for: Comprehensive scoping review on adherence to 24-hour movement guidelines and socioeconomic indicators in children and adolescents
Source: PLoS One. 2025 Apr 17;20(4):e0321103. doi: 10.1371/journal.pone.0321103 (PMC12005491; doi:10.1371/journal.pone.0321103)
Supplement: S1 Table — (PDF) [file pone.0321103.s001.pdf]

Study location	Year of data collection	Study design	Average age or age range (years)	Sample size (% male/female)	Study objective	Author and year of study
Saudi Arabia	2020	Cross-sectional	8.5 ± 1.85 years	1.021 (39.6%; 60.3%)	To investigate the impact of the COVID-19 outbreak on 24-hour movement behaviors among Saudi children aged 6–12 years.	[20]
China	2017	Cross-sectional	13.75 ± 2.61 years	114.072 (49.18%; 50.82%)	To investigate the prevalence of compliance with 24-hour movement guidelines, its correlates, and their relationships with body mass index among children and adolescents in China.	[21]
Mozambique	2017-2018	Cross-sectional	10.1 ± 0.8 years	683 (47.1%;52.9%)	To compare the prevalence of compliance with 24-hour movement guidelines and examine correlates of compliance with the guidelines in a sample of urban and rural Mozambican schoolchildren.	[23]
New Zealand	2009	Longitudinal	7.8± 0.24 years	623 (48.5%; 51.5%)	Describe the 24-hour time-use behaviors of children, both in terms of activity intensity and type of activity; examine the differences in 24-hour time-use behaviors across different sociodemographic groups, and determine adherence to the individual and combined 24-Hour Movement Guidelines for children.	[5]
Republic of Korea	2013 a 2018	Cross-sectional	14.83 ±1.59 years	372.433 (52.1; 47.9%)	To examine the 6-year trend and intersectional correlates of adherence to the 24-Hour Movement Guidelines in a nationally representative sample of Korean adolescents.	[24]
United Kingdom	2015 a 2016	Cross-sectional	14 years	3.899 (n/c)	To examine the associations between sleep, physical activity, and screen time among 14-year-old adolescents in the UK.	[25]
United States	2012 a 2014	Cross-sectional	6.5 ± 1.1 years	1.192 (50.0%; 49.0%)	To examine the proportion of children aged 5 to 8 in the U.S.-affiliated Pacific region who meet the Asia-Pacific 24-hour movement guidelines for physical activity, sleep, and screen time, and to	[26]

					analyze the variation across sociodemographic factors and adiposity measures.	
Brazil	2013 a 2014	Cross-sectional	14.4 ± 1.59 years	58.535 (43.9%; 56.1%)	To assess the prevalence of adherence to guidelines for physical activity, screen time, and sleep duration, as well as potential correlates of movement behaviors, in a large national sample of Brazilian adolescents.	[27]
Germany	2021-2022	Cross-sectional	13.1 ± 1.7 years	7.433 (50.3%; 49.8%)	To examine the relationship between regional socioeconomic deprivation and adherence to 24-hour movement guidelines among children and adolescents.	[28]
China	2019	Cross-sectional	13.6 ± 2.6 years	2614 (4.2%; 52.8%)	To investigate the prevalence of adherence to 24-hour movement guidelines and its sociodemographic correlates in youth from low- and middle-income families in Nanjing, China.	[22]

n/c: nothing to report; %: percentage.
