# Supplementary material for: Comprehensive scoping review on adherence to 24-hour movement guidelines and socioeconomic indicators in children and adolescents
Source: PLoS One. 2025 Apr 17;20(4):e0321103. doi: 10.1371/journal.pone.0321103 (PMC12005491; doi:10.1371/journal.pone.0321103)
Supplement: S2 Table — (PDF) [file pone.0321103.s002.pdf]

Tests/instruments used to assess 24h MB	Classification used for 24h MB	Socioeconomic indicator	Statistical analysis used in the simultaneous combination of variables	Results	Reference
Self-reported by questionnaire	DM and 2020 World Health Organization Guidelines on Physical Activity and Sedentary Behavior. [2,52,53]	Region of residence, parental education (elementary school, high school, bachelor's degree, master's degree, doctorate) and family income (0 to 20.000 or more)	Spearman correlations	Parental education (r = −0.14, p < 0.0001) and income (r = −0.10, p < 0.0001) levels were weakly associated with 24hMC. Adherence to 24hMC decreased as parental education and income levels increased.	[20]
Self-reported by questionnaire	DM [35]	Place of residence (urban or rural), family composition (only child, two children or more), parental education (low education and high education;) and family income per person	Generalized linear models	Regarding the location of residence (reference category = rural. Other categories: urban), it was found that male adolescents living in urban areas were more likely (OR: 1.11; 95% CI: 1.02–1.21) to meet the 24-hour movement guidelines compared to adolescents of both sexes living in rural areas. Regarding parental education (reference category =	[21]

		annually (<9000 or <100000)		low education. Other categories = high education level), those whose parents had a higher level of education were more likely to meet the 24-hour MB guidelines (OR: 1.23; 95% CI: 1.11–1.35). Regarding family income (reference category = <9000. Other categories 9001-30,000; 30,001-100,000 and >100,000), male adolescents from families with the second highest (30,001–100,000) and the highest (>100,000) income had greater chances of meeting the 24h MB guidelines (OR for the second highest: 1.12, 95% CI: 1.00–1.25; OR for the highest: 1.46, 95% CI: 1.26–1.68).	
Sleep and physical activity: Actigraph GT3X accelerometers. Recreational screen	n/c	Parental education (<high school, high school/some college, or bachelor's/graduate degree), school	Multilevel multivariate logit models	For school location (reference category = rural. Other categories: urban), adolescents from urban schools were less likely to meet the 24h MB guidelines (OR: 0.21;	[23]

time was self-reported by questionnaire.		location (urban or rural), number of televisions in the household (0 or 1 or ≥ 2), number of functional cars in the household (0 or 1 or ≥ 2), number of siblings per participant (≤2 or ≥ 3), neighborhood crime rate (“disagreed/strongly disagreed” and “agreed/strongly agreed”), mother's and father's employment status (≤15 h/week or >15 h/week).		95% CI: 0.09–0.52) when compared to those from rural schools. Parental education (reference category = < than high school. Other categories: high school/some college) (OR: 0.37; 95% CI: 0.16–0.87) was a significant correlate of meeting the 24h MB guidelines.	
The Axivity AX3 accelerometer was used to assess 24-hour screen time behaviors. Screen time was self-reported using a questionnaire	New Zealand 24-Hour Movement Guidelines for Children	Household annual income (< or up to 150,000), place of residence (urban and rural) and New Zealand deprivation index, family socioeconomic status,	Chi-square tests and Fisher’s exact test.	Adherence to 24-Hour MB guidelines increased as maternal education levels increased (p = 0.017).	[5]

addressed to the child or adolescent's mother.		maternal education level (no secondary school qualification, secondary school/National Certificate of Educational Achievement, diploma/trade certificate, bachelor’s degree, tertiary diploma), weekly working hours (15, 15–30, 30–40 and ≥ 40 hours), and family structure (single parent, both parents, parent(s) with extended family, or parent(s) living with non-relatives)			
Sleep and physical activity and recreational screen time were self-	DM [2]	Economic status (low, middle or high), social class (set of parental education levels and academic	Multiple logistic regression	Regarding socioeconomic status (reference category = female + low. Other categories: Female + medium, Female + high, Male + low, Male + medium, Male +	[24]

reported by questionnaire.	performance) and parental education (<undergraduate and ≥ undergraduate, with the reference category being female with parental education <undergraduate)	high), during weekdays and weekends male adolescents classified as low (OR: 1.13; 95%CI: 1.09–1.17), medium (OR: 1.18; 95%CI: 1.15–1.22) and high (OR: 1.08; 95%CI: 1.05–1.12) status were associated with compliance with the 24hCM movement guidelines. Females classified as having high family economic status were more likely to meet the 24h MC guidelines compared to those with low economic status on weekdays (OR: 1.60; 95% CI: 1.02–2.50) (reference category = female + low. Other categories: Female + middle, Female + high, Male + low, Male + middle, Male + upper class). For maternal and paternal education, it was found that male adolescents whose maternal or paternal education was less than higher education and male adolescents whose maternal or paternal education was equal to or	

				greater than higher education were more likely to meet the recommendations of all 24h MB compared to female adolescents whose maternal or paternal education was less than higher education, both on weekdays and weekends (reference category = Female + < post-secondary. Other categories: Female + ≥ post-secondary, Male + < post-secondary and Male + ≥ post-secondary).	
Sleep duration and screen time were self-reported, and physical activity was assessed using the GENEActiv wrist-worn accelerometer (Activinsights Ltd).	n/c	Family income tertile (low, medium and high)	Logistic regression	Regarding income, females with the highest income tertile were more likely to meet 24h CM guidelines (OR: 2.13; 95% CI: 1.28-3.54), when compared with those in the lowest income tertile (reference category = lower. Other categories = medium and high).	[25]

Sleep duration and physical activity were measured using an Actical accelerometer (Z series, Philips Respironics Inc). Screen time was self-reported by questionnaire by caregivers of the children and adolescents.	Asia-Pacific 24-Hour Movement Guidelines [54]	Jurisdiction income (high, upper-middle, lower-middle, and low income levels), caregiver education (incomplete high school, high school or General Educational Development degree, and higher education), and annual household income (below $10,000, $10,000 to $35,000, and above $35,000)	Generalized linear mixed models	Regarding jurisdiction income (reference category = high. Other categories: upper-middle and lower middle), those with lower jurisdiction income were more likely to meet the 24h MB guidelines when compared to those in high-income jurisdictions (OR: 2.33; 95% CI: 1.48-3.67). Regarding parental education (reference category = 11th grade or less. Other categories: high school or GED and attended college), those whose parents had higher education were less likely to meet the 24h MB guidelines when compared to those who did not complete high school (OR: 0.52; 95% CI: 0.33-0.83 and OR: 0.49; 95% CI: 0.32-0.76). Regarding income (reference category = below $10,000. Other categories: $10,000 to <$35,000 and $35,000 and more) those with higher income were less likely to meet	[26]

				24h CM guidelines (OR: 0.60; 95% CI: 0.39–0.92).	
Sleep and physical activity and recreational screen time were self-reported by questionnaire.	Sleep time [31], screen time [30], and physical activity [29].	Socioeconomic status (1: low, 2: medium, 3: high) and region of residence (north, northeast, central-west, southeast, south)	Poisson regression	Regarding region of residence (reference category = North. Other categories: Northeast, Central-West, Southeast, South), adolescents who lived in less developed regions showed greater adherence to the 24hCM guidelines, when compared to those who live in more developed regions of Brazil.	[27]
Sleep duration, physical activity, and screen time were self-reported by questionnaire.	DM [2]	Regional socioeconomic deprivation (regional socioeconomic indicators in the areas of income, education and occupation), classified into quintiles (quintile 1, the one with the lowest socioeconomic deprivation, and quintile 5, the one with the highest	Multilevel logistic regression	Socioeconomic deprivation was divided into quintiles, with quintile 1 having the lowest socioeconomic deprivation and quintile 5 having the highest socioeconomic deprivation (reference category: quintile 1). Adolescents in quintile 5 (highest socioeconomic deprivation) were less likely to meet the 24h MC guidelines when compared to adolescents from richer regions (OR: 0.49; 95% CI: 0.28; -0.03], p = 0.010).	[28]

		socioeconomic deprivation).			
Sleep and physical activity and screen time were self-reported by questionnaire.	DM [2]	Place of residence (urban or rural); education of both parents (less than college or university; college or university; graduate degree); and household wealth based on annual per capita income (<9000; 9000-30,000; >30,000-100,000; >100,000).	Generalized linear models	Regarding place of residence, (reference category: rural. Other categories: urban) those who live in rural areas are more likely to meet the 24h CM guidelines (OR: 2.5; 95% CI: 1.5-4.1)	[22]

24h MB: 24-hour movement behavior; DM: Canadian 24-hour Movement Guidelines; <: less than; >: greater than; ≤: less than or equal; ≥: greater than or equal; US$: dollar; r: correlation; OR: odds ratio; 95%CI: confidence interval; p: p-value.
